# Beam commissioning of a new compact scanned proton therapy system with four different dose calculation algorithms

**DOI:** 10.1002/acm2.70433

**Published:** 2025-12-19

**Authors:** Ryo Tanokura, Masato Horita, Makoto Matsumoto, Akari Miyahara, Shinji Furukawa, Yasushi Ido, Akira Matsumoto, Shinichi Ogawa, Wataru Makino, Yoshihito Matsui, Yuki Tominaga, Takahiro Kato, Nobukazu Fuwa

**Affiliations:** ^1^ Department of Radiological Technology Central Japan International Medical Center Minokamo Gifu Japan; ^2^ Department of Radiation Oncology Central Japan International Medical Center Minokamo Gifu Japan; ^3^ Department of Radiotherapy, Medical Co. Hakuhokai Osaka Proton Therapy Clinic Konohana‐ku Osaka Japan; ^4^ Medical Physics Laboratory Division of Health Science Graduate School of Medicine The University of Osaka Suita‐Shi Osaka Japan; ^5^ Department of Radiological Sciences, School of Health Sciences Fukushima Medical University Sakae‐machi Fukushima Japan

**Keywords:** commissioning, dose calculation algorithm, pencil beam scanning, proton therapy, treatment planning system

## Abstract

**Background:**

The Central Japan International Medical Center (Gifu prefecture, Japan) as the 20th Proton Therapy Institution in Japan, has newly installed a compact scanned proton therapy system, ProBeam 360°™. We installed two commercial treatment planning systems (TPSs): Eclipse and RayStation. No commissioning results have been reported between Varian's newest compact proton therapy system and the two TPSs.​

**Purpose:**

We identified the optimal dose calculation algorithm for clinical use by evaluating the dose agreements between four dose calculation algorithms provided by two TPSs and the measured values using our new machine.

**Methods:**

The two TPSs each had a pencil beam algorithm (PBA) and Monte Carlo (MC) dose engines (four in total). Validation plans were created for 26 box targets (with and without a 50‐mm range shifter) in a homogeneous phantom optimized using Eclipse. We created three realistic clinical plans consisting of 42 beams in mock patients with prostate, head and neck, and C‐shaped targets. We validated 12 heterogeneous plans for the lung and bone regions using all three optimizations and four dose algorithms with the TPSs. Measurements were performed on the lateral profiles at the isocenter plane. The calculated doses for each algorithm were compared with the measured doses.

**Results:**

Each dose algorithm achieved an average gamma score of at least 95.6 ± 5.0% or higher (RayStation PBA) at 3%/3 mm in the box plans. For the patient and heterogeneous plans, the average gamma score exceeded 95% for only two MC algorithms. RayStation MC showed the highest dose calculation accuracy in both homogeneous and heterogeneous regions.

**Conclusions:**

All calculated doses of the box plans were in good agreement with the measurements, except for the RayStation PBA. For clinical use, RayStation MC is the preferred option for both homogeneous and heterogeneous cases.

## 1 INTRODUCTION

Pencil beam scanning (PBS) proton therapy is one of the most advanced technologies in radiation therapy owing to its superior physical characteristics.^1^ The number of PBS facilities is increasing worldwide, and many vendors have developed treatment devices with unique features, such as IBA, Hitachi, Mitsubishi, Sumitomo, and Mevion.^2^, ^3^, ^4^, ^5^, ^6^ Each system undergoes full commissioning before clinical use to ensure safe proton delivery.

Varian introduced spot‐scanning systems, such as the multi‐room‐type ProBeam, followed by the single‐room‐type ProBeam Compact.^7^, ^8^ In March 2024, Central Japan International Medical Center (CJIMC; Gifu prefecture, Japan) as the 20th Proton Therapy Institution, installed a ProBeam 360°, a state‐of‐the‐art Varian machine, and began treatment for prostate cancer. This machine was the first of its kind to be introduced in Japan and the second in the world. This machine was further downsized compared with the previous two models, ProBeam and ProBeam Compact, and the source‐to‐axis distance, irradiation field, and energy range were reduced and updated accordingly. Because no commissioning data for ProBeam 360° were available, we assessed whether our validation results aligned with those in prior ProBeam reports. CJIMC installed two types of treatment planning systems (TPSs): Eclipse ver. 16.1 (Varian Medical System, Palo Alto, CA, USA) and RayStation 10A (RaySearch Laboratories, Stockholm, Sweden) at the beginning of the project.^9^, ^10^ Each TPS has two dose calculation algorithms (four in total). Although several algorithms for the two TPSs have been commissioned, no report has compared the four algorithms simultaneously.^11^, ^12^, ^13^ We believe that evaluating the agreement between the measurements and calculations of the four algorithms and identifying the characteristics specific to each calculation will provide useful information for future facilities installing PBS machines and their respective TPSs.

Therefore, we performed clinical beam commissioning of the newly introduced ProBeam 360° at CJIMC. By comparing the obtained modeling data and beam commissioning results with reports on prior machines, a comprehensive evaluation was performed to determine whether the accuracy was acceptable for clinical applications.

## 2 THEORETICAL FRAMEWORK

### 2.1 Our proton therapy system

We introduced a new scanned proton therapy system developed by Varian.^14^ The accelerator of the ProBeam 360° is an isochronous superconducting cyclotron, which is capable of a maximum beam intensity of up to 800 nA (1–2 nA for patient treatment) and is equipped with hardware that allows for FLASH irradiation.^15^ The commissioning in this study was limited to regular PBS beams. The cyclotron produced protons with a fixed energy of 226 MeV. Then, the energy selection system, consisting of the degrader, was adjusted to the appropriate energy in the range of 60–224.3 MeV. The treatment room was equipped with a rotating gantry that could irradiate from 360° (± 190°) with a rotation speed of 6°/s. For uniform irradiation of areas shallower than the minimum energy, range shifters (RSs) made of polycarbonate PC 1000 (Lexan) of 20, 30, and 50 mm (23, 34, and 57 mm in water equivalent thickness) are available. The treatment nozzle position range was 0–420 mm from the isocenter. The maximum irradiation field is 25 × 25 cm^2^, which is smaller than that of previously released ProBeam systems.^7^, ^16^ The smaller beamline and treatment nozzle reduced the source‐to‐axis distance to 1340 and 1750 mm in the *x*‐ and *y*‐directions, respectively.

The patient positioning system was a dedicated six‐axis carbon couch system. The drive limits of the rotation system were ±3°, ±3°, and ±95° for roll, pitch, and rotation, respectively. The kV Imager and CBCT system were integrated into the gantry. The kV Imager performed simultaneous imaging of 0.4–1000 mAs values in the frontal and lateral views at 40–140 kV. The CBCT system uses tube voltages of 80–140 kV and can perform one rotation (360°) and a half rotation (193°). As the gantry rotated at a speed of 6°/s, the acquisition times were 60 and 32 s, respectively. The thickness of each slice was 2 mm.

### 2.2 Four dose calculation algorithms in the two TPSs

Both TPSs are commercial systems: Eclipse with proton convolution superposition (PCS), AcurosPT (AXPT), RayStation with PBA, and Monte Carlo (MC) calculation algorithms. In this study, the dose validation of the four dose calculation algorithms was performed using the same plan. The optimizer of Eclipse was the non‐linear universal proton optimizer (NUPO).^9^ The PCS and RayStation PBA (RayPBA) were modeled using a double Gaussian model.^17^, ^18^ These calculations are based on analytical methods using beam modelling data acquired in water, enabling high‐precision calculations of dose distribution in homogeneous tissues with densities close to that of water within a short time. However, the dose to heterogeneous regions is considered inferior dose calculation accuracy.^19^, ^20^

AXPT solves the Boltzmann transport equation using the MC algorithm. The AXPT simplifies proton transport using continuous slowing down, multiple Coulomb scattering (MCS) with Gaussian approximations, and reduced modeling of non‐elastic nuclear products, thereby balancing computational efficiency with clinically acceptable accuracy.^13^ These include the introduction of approximate models (continuous deceleration approximation: simplification of continuous energy loss calculations using continuous slowing down [CSD], Gaussian distribution approximation of multiple Coulomb scattering, consideration of calculations for limited inelastic reactivity of biological materials), control of probabilistic processing (modeling of energy straggling, deposition of residual energy), parallel processing (acceleration through parallel computing using graphical processing units [GPUs]), and early termination of calculations through monitoring of statistical uncertainty on a batch basis.

RayStation MC (RayMC) is a variance‐reduced Monte Carlo engine tailored for clinical transport with simplified secondary‐particle handling.^10^ By implementing simplified transport processing (ignoring secondary particles, neutrons, gamma rays, and delta electron formation), optimizing transport mechanisms (using the random hinge method), parameterizing the model (Bethe–Bloch equation parameterization model), and optimizing statistical error processing, the model does not simply implement full MC but rather simplifies and optimizes transport processing and physical processes in a manner suitable for clinical applications. These MC algorithms provide high accuracy in dose calculations for plans in heterogeneous regions but are time consuming.^21^, ^22^ Both versions of the TPS used in this study can perform MC calculations using GPUs.^23^ Central processing unit (CPU) calculations have already achieved short computation times, and GPU calculations have further accelerated the process. GPU acceleration yielded ∼10–20× faster calculations than the CPU via per‐particle parallelization.^10^

## 3 MATERIALS AND METHODS

This section mainly describes the registration of beam data necessary for performing dose calculations using the two TPSs (Sections 3.1–3.3), the method for creating a commissioning plan (Sections 3.4–3.6), and the measurement and evaluation methods for verifying the dose using the commissioning plan (Section 3.7).

### 3.1 Beam modeling data

**FIGURE 1:**
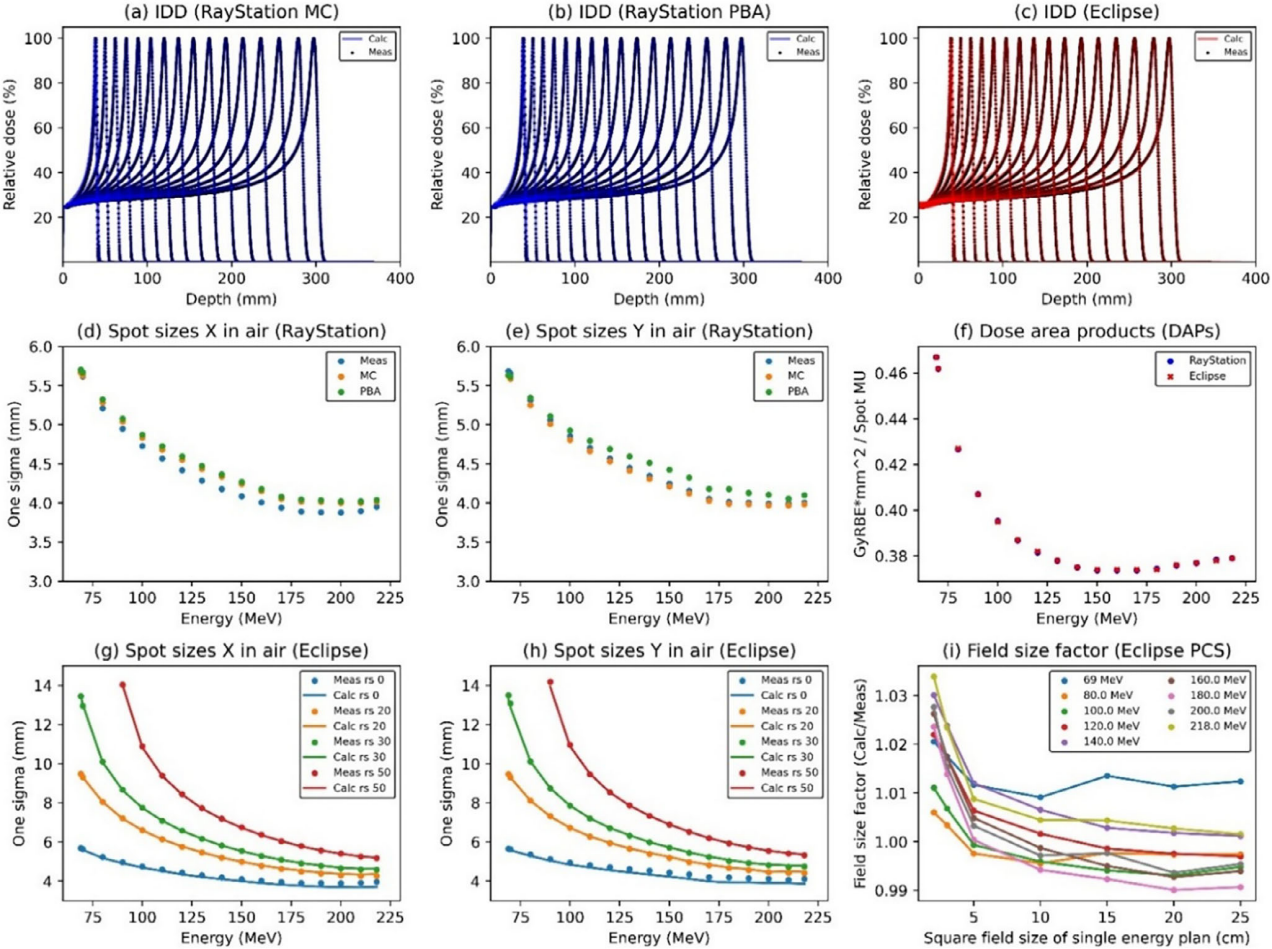
Comparisons between the measured and modelled beam data in two different treatment planning systems of RayStation and Eclipse for (a–c) IDD, (d, e, g, h) spot sizes, (f) dose area products, and (i) field size factor.IDD, integrated depth dose; MC, Monte Carlo algorithm; PBA, pencil beam algorithm; Meas, Measured data; Calc, Calculated data; rs, range shifters.

The data required for PBS beam modeling with both TPSs were: (1) integrated depth doses (IDDs), (2) spot profiles in air, and (3) absolute dose calibrations at a depth of 20 mm in water (Figure 1 and Table S1).^7^ All measurements were performed at a gantry angle of 0°. IDD and spot profile measurements were performed for single‐pencil beam spots. The data acquired for modeling ranged from 70 to 210 MeV at 10 MeV intervals, with minimum and maximum energies of 69 and 218 MeV, respectively, for a total of 17 energies. The IDDs were measured using a 3D water phantom (MP3‐M, PTW, Freiburg, Germany) and an 8.16‐cm diameter parallel ionization chamber (Type 34070, Bragg Peak Ionization Chamber, PTW, Freiburg, Germany). In Eclipse, the IDD curve must be registered as an absolute dose because the system has no feature for Gy per monitor unit (MU). In RayStation, the IDD is registered as a relative curve, with a separate feature to enter the Gy per MU at a 20 mm depth, so the DMU can be updated without changing the IDD curve. These TPS‐specific requirements differ; however, both systems ultimately yield the same absolute IDD for the measured data. The measured IDDs were compared with the calculated range for each energy obtained from the National Institute of Standards and Technology Proton Stopping Power and Range (NIST) Continuous Slowing Down Approximation (CSDA) range.^24^ The spot profiles were measured using a 2D scintillator detector with a CCD camera (XRV‐2000 Falcon; Logos Systems International, Scotts Valley, CA, USA).^25^ The spot profiles were measured at the isocenter without RSs for all 17 energies and additional planes of −200, −100, +100, and +200 mm from the isocenter, as well as modeling of the other Varian machines.^17^ Spot profiles were added to the Eclipse PCS registration data using three different RSs values (20, 30, and 50 mm). The air gap (distance between the scintillator surface and RSs) was set to 30 mm (minimum possible setting) for all conditions, a 50 mm RS was used down to a minimum of 90 MeV, and all 17 energies were measured using the other RSs.

Beam sizes were entered into the TPS from line profiles in the in‐line and cross‐line directions obtained from the .tiff format images using in‐house analysis software. The average one‐sigma spot sizes at the isocenter plane between the measured and modeled data were compared for both line directions. The absolute dose calibration plan consisted of 41 spots, each vertically and horizontally spaced by 2.5‐mm spacing, creating a single energy field of 100 × 100 mm^2^. The plans were measured using a calibrated parallel‐plate ionization chamber (Advanced Markus Chamber, Type 34045; PTW, Freiburg, Germany) at a water depth of 20 mm.

### 3.2 Registration of field size correction factor that exists only in eclipse

In this version of Eclipse, only the PCS can register the power output at different irradiation fields for each energy and correct the output factor (called the field size factor) by its ratio to the calculated value.^13^ In practice, this factor is corrected by the number of spots formed at regular intervals that form the irradiation field for each energy. Thus, in this study, the correction was measured before commissioning and registered in the TPS. Single‐energy irradiation of 2, 3, 5, 10, 15, and 20 cm square fields was created for nine energies, including 20 MeV increments and minimum and maximum energies (69 and 218 MeV) in the 80–200 MeV range. The spot spacing was 5.0 mm for all energies, based on a previous study that performed a validation similar to the field size factor.^26^, ^27^ The flatness of the 5‐mm spot spacing at a depth of 20 mm and the rate of variation of the measured values were checked before the factor measurement to confirm that the plan was acceptable. We derived field‐size factors as measured/TPS and registered them to PCS as output corrections.

### 3.3 Computed tomography to the relative stopping power and mass density table

To perform dose calculations for computed tomography (CT) images, it is necessary to register the relative stopping power and mass density for the CT values (Hounsfield Units, HUs).^12^ In Eclipse, PCS and NUPO use relative stopping power, whereas AXPT uses mass density for dose calculation. In contrast, RayStation can perform dose calculations using either relative stopping power or mass density. In this study, the relative stopping power was used in RayStation to unify Eclipse's PCS and RayStation's tables. The method used for the relative stopping power conversion was the stoichiometric calibration proposed by Schneider et al.^28^ The phantom used to create the CT table was an Electron Density Phantom Model 062 M (CIRS, Norfolk, VA, USA). Nine different rods were imaged using a CT system to obtain the HU and suppression ratios. Finally, the fitting and approximate relative stopping powers were derived for six different HUs: −1000, −150, −70, 23, 60, and 2000.

### 3.4 Commissioning plans for box‐shaped targets

For basic validation, box‐shaped targets of various sizes were created in a virtual phantom assigned a homogeneous water density (1.0 g/cm^3^) based on the guidelines for scanned proton therapy systems.^29^ The size of the irradiation field was selected to range from 3 × 3 to 15 × 15 cm^2^, with a modulation width (spread‐out Bragg peak [SOBP] width) of ≤10 cm (Table S2). All plans were created at a gantry angle of 0° with the isocenter positioned at the center of the target. In this study, robust optimization was not used in any of the verification treatment plans described in Sections 3.4–3.6. Plans without RS (Box plan) and with a 50‐mm thick RS (BoxRS plan) were optimized for 14 and 6 different target geometries, respectively. In the BoxRS plans, all optimizations were performed by manually setting a 50‐mm‐thick RS to be used during planning. The total number of BoxRS plans was 12 because two BoxRS plans were created with air gaps of 5 and 15 cm. All plan optimizations were performed using the Eclipse NUPO algorithm. Final calculations after optimization were performed for all dose algorithms, including AXPT, PCS, RayMC, and RayPBA. In this study, the optimization was unified into one type; as a result, the only variables were the calculation results from the four different algorithms. This made it possible to evaluate the goodness of the four different dose calculation results with only one type of measurement result from a single plan. Based on a previous report, the statistical uncertainty in both AXPT and RayMC calculations was set to 0.5%.^30^

### 3.5 End‐to‐end tests for patient mock plans

To validate the field of simulated clinical plans that closely resemble actual patient conditions, prostate, head and neck (HN), and C‐shape plans were selected from the American Association of Physicists in Medicine Task Group 119 phantom (TG‐119 phantom).^31^ The optimization and dose calculation were performed as described in Section 3.4. Each plan used 2–3 beams and was optimized to meet dose constraints using the target and organs at risk contours defined in previous studies (Figure S1 and Table S2) and both single‐field optimization (SFO) and multi‐field optimization (MFO).^32^ In this study, we refer to the beams optimized by SFO and MFO as SFO and MFO beams, respectively. Four different plans were created for these two targets (HN and C‐shape plans), including the RS plans (20‐, 30‐, and 50‐mm thicknesses). Therefore, the HN and C‐shaped plans contained 24 and 16 beams, respectively (Table S2). In total, 42 mock patient beams were calculated with a gantry angle of 0° in a homogeneous phantom for validation plans, and the depth doses were measured for 12 of these beams (Table S2). For the HN and C‐shaped plans, MFO beams were selected to measure the depth doses with no RS and maximum RS (50‐mm thickness).

### 3.6 Commissioning plans for a heterogeneous phantom

To validate the heterogeneous plans, four different rods were inserted, and a cheese phantom was used, as described in a previous study.^33^ The target volume was 9 × 9 × 4 cm^3^, and the four rods inserted into the target consisted of lung1, bone, water, and lung2 with densities of 0.490, 1.139, 1.000, and 0.290 g/cm^3^, respectively (Figure S1D, E). The interior of the measurement device exhibited significant density variations between regions containing ionization chamber dosimeters and those without, potentially introducing uncertainty in the dose calculations within this area (Figure S1D, E). Therefore, based on previous reports, this region was replaced with water density for treatment planning.^34^, ^35^ Verification of the heterogeneous phantom was performed not on the detector itself, but using four rods of different densities inserted in front of the measurement cross‐section. Therefore, even if the detector itself was density‐replaced, we considered that poor calculation accuracy in the heterogeneous region would alter the agreement of the dose distribution. Treatment plans were recalculated using all four computational algorithms (PCS, AXPT, RayPBA, and RayMC) for each of the three optimization algorithms (NUPO, RayPBA, and RayMC); therefore, three plans were created for each optimization algorithm (Figure S1 and Table S2; 12 plans in total).

### 3.7 Measurements and evaluations

The measurements for beam commissioning included the depth doses and 2D dose distributions at the isocenter plane. Depth doses were measured using a PinPoint 3D ionization chamber (Type 31022, PTW, Freiburg, Germany), an electrometer (UNIDOS Tango, PTW, Freiburg, Germany), and a 3D water phantom (MP3‐M, PTW Freiburg, Germany). The depth doses were measured at the central axis for the 22 beams of the box‐shaped plans and 12 beams of the TG‐119 plans (Table S2). The measured doses were analyzed using the global gamma index with criteria set at 3%/3 mm (10% threshold), employing custom scripting based on the Pymedphys library.^36^, ^37^

Lateral dose profiles were measured using a cross‐calibrated 2D ion chamber detector array (2D‐array, OCTAVIUS 1500XDR, PTW, Freiburg, Germany) and a solid water phantom (Plastic Water, CIRS, Norfolk, VA, USA). We measured the 2D dose distribution in the isocenter plane. Therefore, the numbers of measurement planes were 14, 12, and 42 for the box‐shaped plans, box‐shaped plans with RS, and TG‐119 plans, respectively (Table S2). The measured 2D dose distributions were analyzed using the global gamma index of 2%/2 mm and 3%/3 mm with a 10% threshold.

Absolute doses were measured at the center of the SOBP for all plans using a 2D‐array. The isocenter point doses were obtained using an ionization chamber dosimeter placed at the center of the 2D‐array. The dose differences between the measured ($D_{m}$) and calculated doses ($D_{c}$) were calculated using Equation (1).

(1) $$Dose\;differences=\frac{D_{m}-D_{c}}{D_{c}}\times 100\;\left(\%\right)$$

For the heterogeneous plan, measurements were performed once for each of the three optimized plan beam MUs. Analysis was performed on the agreement between the calculations and measurements owing to differences in the optimization and final calculation algorithms for a 3%/3‐mm gamma analysis and absolute dose difference. For the gamma analysis, the tolerance level of gamma scores was determined to be at least 90% at 3%/3‐mm based on a previous report.^38^ For the absolute dose difference, the points measured near the center of the rod were selected for three different rods (one bone and two lungs), excluding the isocenter (homogeneous region) and water, to check for dose agreements. Furthermore, to compare the optimization and final calculation times of the four different dose calculation algorithms, the time required for the planning of the heterogeneous plan was obtained using the time measurement tool in the TPS.

## 4 RESULTS

### 4.1 Modeling data validations

Figure 1 and Table S1 show the measurement and TPS modeling results for the IDD (Figure 1a–c), spot sizes x (Figure 1d–g), spot sizes y (Figure 1e–h), and absolute dose calibrations (Figure 1f). The measured IDD range error agreed within ±0.70 and ±0.21 mm for the NIST approximation (CSDA ranges) and TPSs modeling data, respectively. The one‐sigma spot sizes in air (69.0–218.0 MeV energy ranges) were 3.95–5.67 mm and 4.01–5.69 mm in the *x* (cross‐line) and *y* (in‐line) directions, respectively. The spot size differences were within ±0.2 mm for both TPSs. We used the same measurement sets for overlapping items; thus, differences did not arise from input data but from modeling accuracy itself. Figure 1i shows the field‐size factor results for nine different energies in the Eclipse PCS. For fields smaller than 3 × 3 cm^2^, the factor exceeded 1.000 for all energies, with the largest factor being 1.034 at 218 MeV, where a correction of >3% was applied. For irradiation fields larger than 10 × 10 cm^2^, correction factors smaller than 1 were adopted for more than half of the energies, with the smallest factor being 0.990 at 180 MeV.

### 4.2 CT to relative stopping power and mass density table

Figure S2 shows the CT to (a) relative stopping power tables, and (b) mass density table. Eclipse and RayStation use the same relative stopping power tables. Dose calculations for the heterogeneous phantoms were performed using these tables.

### 4.3 Validation plans for box‐shaped targets

**FIGURE 2:**
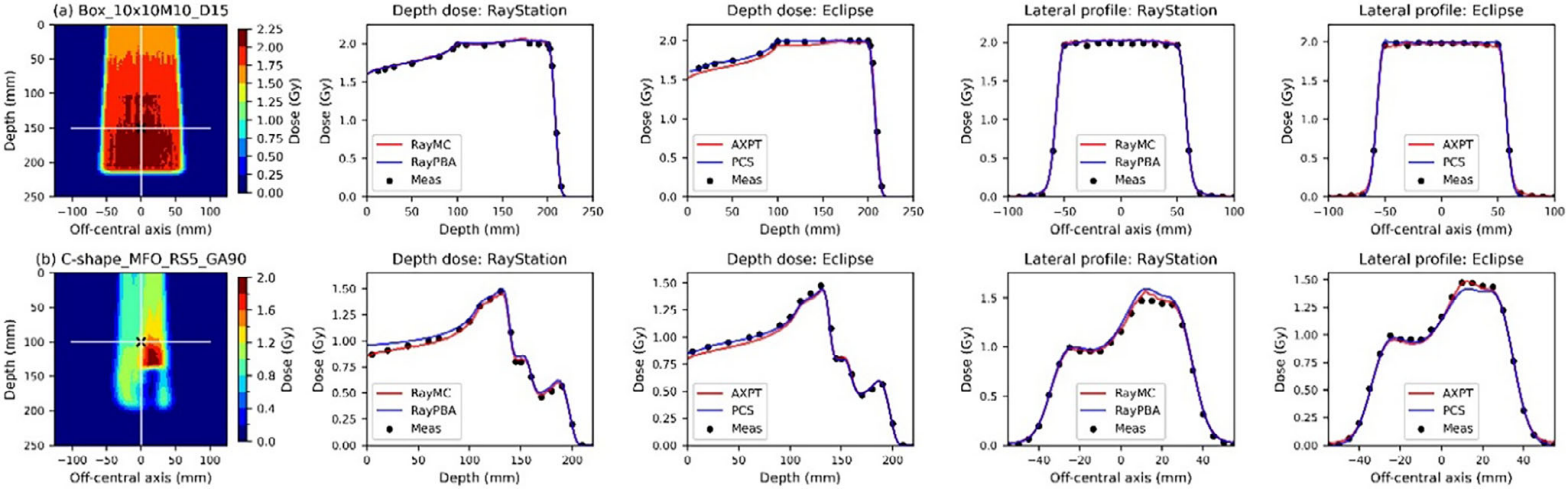
Two representative validation plan dose distributions (first column), depth doses of RayStation (second column) and Eclipse (third column), and lateral profiles of RayStation (fourth column) and Eclipse (fifth column) for (a) box without RS and (b) C‐shape MFO plans. The black cross points indicate the isocenter. The white lines in the figure indicate the measured cross‐sections for the depth doses and lateral profiles of the respective plans. RS, RS; HN, head and neck; MFO, multi‐field optimization; SFO, single‐field optimization; Meas, Measured data; RayMC, RayStation Monte Carlo algorithm; RayPBA, RayStation pencil beam algorithm; AXPT, Eclipse AcurosPT algorithm; PCS, Eclipse proton convolution superposition algorithm.

**FIGURE 3:**
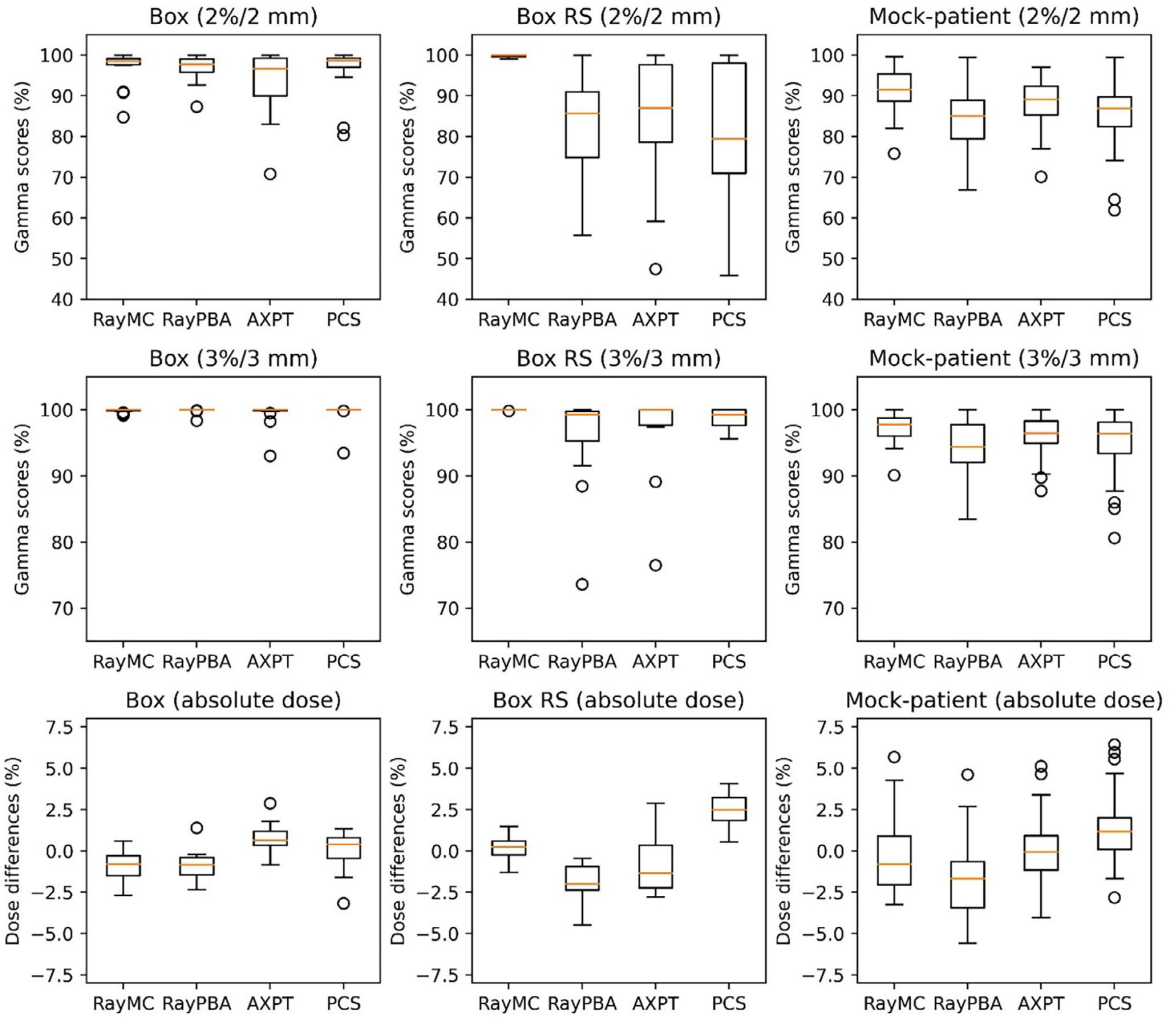
Boxplots of gamma scores in 2D dose measurements at 2%/2 mm (first row), 3%/3 mm (second row), and point doses at the isocenter planes (third row) for the four dose calculation algorithms at different depths in terms of Box, BoxRS, and mock patient plans. The red lines in the figure indicate 90% and ± 5% tolerance values for the gamma scores and point doses, respectively.RayMC, RayStation Monte Carlo algorithm; RayPBA, RayStation pencil beam algorithm; AXPT, Eclipse AcurosPT algorithm; PCS, Eclipse proton convolution superposition algorithm; RS, Range shifter 2 cm or 3 cm, RS5, Range shifter 5 cm; noRS, Without range shifter.

Figure 2 shows the example measurement results for the (a) box‐shaped plan. Figure S3 shows the verification results for the remaining three representative plans using the same display format as Figure 2. The beam shown in Figure S3A contains a 50 mm RS. The calculated values are shown separately for each TPS, with the MC and PBA results shown in red and blue, respectively. Figure 3 also shows a summary of the gamma scores and absolute dose difference results using boxplots. Figures S4,S5 show scatter plots of the gamma scores and absolute dose difference results based on the measurement depth and irradiation area. Table 1 shows the validation results for the depth doses, 2D dose distributions, and absolute doses. Depth‐dose gamma scores exceeded 95% (3%/3 mm) for all Box and BoxRS plans. However, overestimations and underestimations of the doses were confirmed in the plateau region for RayPBA and AXPT, respectively (Figure 2a, b). The gamma scores at 3%/3 mm for the Box plans in the 2D dose distributions exceeded 99.3 ± 1.8% on average for all four algorithms. For the BoxRS plans, the gamma scores exceeded 95.5 ± 7.5% on average for all four algorithms, while algorithms other than RayMC had minimum values below 90% at 2%/2 mm and 3%/3 mm (Table 1 and Figure 3). The absolute dose differences were within ± 3% for all Box plans, except for one PCS case, and several BoxRS plans exceeded ± 3%. The absolute dose differences exceeding 3% were concentrated at depths below 100 mm (third row in Figure S4). The Eclipse PCS achieved gamma scores and absolute dose differences within acceptable limits for small irradiation fields measuring 3 × 3 cm^2^ (second and third rows of Figure S5).

**TABLE 1:** Validation results for gamma scores of depth doses, 2D dose distributions, and absolute dose differences at the isocenter points.

Plan type	Dose calculation algorithms	Gamma scores for depth doses at 3%/3 mm (Minimum)	Gamma scores for 2D dose distributions at 2%/2 mm (Minimum)	Gamma scores for 2D dose distributions at 3%/3 mm (Minimum)	Absolute dose differences at the isocenter point (Minimum/Maximum)
Cubic targets (Box)	RayMC	99.3 ± 2.0% (93.3%)	96.6 ± 4.3% (84.8%)	99.9 ± 0.3% (99.2%)	−0.8 ± 0.9% (−2.7%/0.6%)
	RayPBA	99.7 ± 1.0% (96.7%)	96.6 ± 3.4% (87.3%)	99.9 ± 0.4% (98.4%)	−0.8 ± 0.9% (−2.4%/1.4%)
	AXPT	95.7 ± 7.1% (76.7%)	93.4 ± 8.2% (70.8%)	99.3 ± 1.8% (93.0%)	0.8 ± 0.8% (−0.8%/2.9%)
	PCS	95.7 ± 5.3% (83.3%)	96.0 ± 6.2% (80.3%)	99.5 ± 1.7% (93.4%)	0.0 ± 1.2% (−3.2%/1.3%)
Cubic targets with range shifter (BoxRS)	RayMC	100.0 ± 0.0% (100%)	99.8 ± 0.3% (99.1%)	100.0 ± 0.0% (99.8%)	0.2 ± 0.7% (−1.3%/1.5%)
	RayPBA	95.2 ± 5.5% (84.2%)	82.3 ± 12.6% (55.7%)	95.5 ± 7.5% (73.6%)	−1.9 ± 1.1% (−4.5%/‐0.4%)
	AXPT	98.7 ± 4.4% (84.2%)	83.5 ± 15.8% (47.5%)	96.6 ± 6.7% (76.5%)	−0.6 ± 1.9% (−2.8%/2.9%)
	PCS	97.5 ± 4.4% (88.2%)	80.4 ± 17.8% (45.9%)	95.8 ± 10.1% (62.6%)	2.5 ± 1.0% (0.5%/4.1%)
Mock patients (AAPM TG‐119)	RayMC	88.9 ± 13.0% (58.3%)	91.6 ± 4.8% (75.8%)	97.5 ± 2.0% (90.1%)	−0.4 ± 2.0% (−3.3%/5.7%)
	RayPBA	85.5 ± 13.0% (58.3%)	84.8 ± 7.9% (66.9%)	94.2 ± 4.1% (83.4%)	−1.7 ± 2.2% (−5.6%/4.6%)
	AXPT	90.1 ± 10.5% (73.7%)	88.5 ± 5.6% (70.1%)	96.2 ± 2.8% (87.7%)	0.2 ± 1.9% (‐4.0%/5.1%)
	PCS	83.1 ± 17.4% (46.1%)	85.6 ± 7.7% (61.9%)	94.9 ± 4.3% (80.6%)	1.3 ± 1.9% (−2.8%/6.4%)
Heterogeneous	RayMC	–		97.8 ± 2.6% (93.5%)	Water: 0.7 ± 2.0% (−2.1%/2.3%) Bone: 0.4 ± 1.2% (−0.9%/2.1%) Lung: 0.7 ± 1.9% (−3.7%/2.3%)
	RayPBA	–		90.1 ± 2.7% (86.2%)	Water: 1.0 ± 1.6% (−0.5%/2.4%) Bone: −1.1 ± 1.8% (−2.5%/1.1%) Lung: 3.9 ± 2.5% (0.1%/7.0%)
	AXPT	–		92.7 ± 8.0% (79.0%)	Water: 0.8 ± 1.3% (−0.8%/2.3%) Bone: 0.2 ± 1.2% (−1.2%/1.7%) Lung: 2.8 ± 5.0% (−3.4%/12.8%)
	PCS	–		78.6 ± 13.2% (61.1%)	Water: ‐0.2 ± 2.3% (−2.9%/2.3%) Bone: 0.4 ± 1.6% (−1.2%/2.6%) Lung: 5.3 ± 5.5% (−2.8%/12.8%)

The absolute dose differences for heterogeneous plans show comparisons between the measurements and calculations at the center points of the water, bone, and lung rods. Abbreviations: RayMC, RayStation Monte Carlo algorithm; RayPBA, RayStation pencil beam algorithm; AXPT, Eclipse AcurosPT algorithm; PCS, Eclipse proton convolution superposition algorithm

### 4.4 Validation plans for mock‐patient targets

Figure 2b shows the measurement results for the example simulated patient plans. The beams in Figure 2b and S3b have a 50 mm RS. The beam angles of Figure 2b and S3 in the TG‐119 phantom were 90°, 0°, and 90° for the (2b) C‐shape MFO, (S3b) HN MFO, and (S3c) prostate plans, respectively. The depth dose of RayPBA resulted in a dose overestimation in the plateau region. In addition, dose underestimation was confirmed in the plateau region for AXPT. Thus, the gamma score for the depth doses worsened because of the difficulty in computationally representing the steep distal dose distribution (Table 1). The gamma scores of the 2D dose distributions at 3%/3 mm were greater than 90% for all the beams in RayMC. These results include both MFO and SFO and are good even for MFO beams with non‐uniformity in the target. For RayPBA, AXPT, and PCS, gamma scores below 90% were limited to depths below 100 mm, particularly for the 50 mm RS plan (second row, Figure S4). The absolute dose differences were within ± 5% error only for the RayMC plans (Table 1).

### 4.5 Validation plans for heterogeneous condition

**FIGURE 4:**
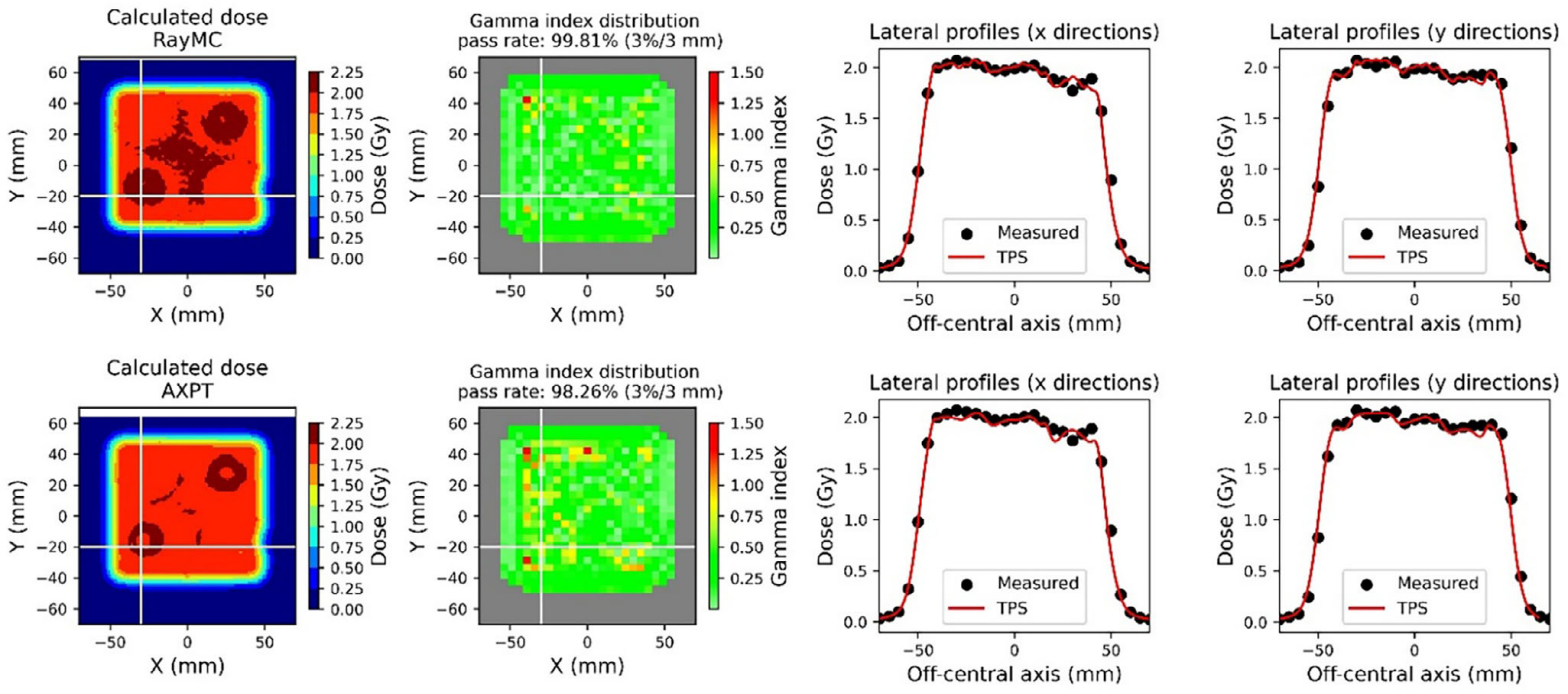
Validation results of heterogeneous plans with NUPO optimization for two different dose‐calculation algorithms (RayMC and AXPT). The white lines in the dose and gamma‐index distributions represent the cross‐sections shown in the lateral profiles (*x*‐ and *y*‐directions).

Figure 4 and Table 1 show the results of the gamma analysis. In the 2D dose distribution, RayMC was the only calculation algorithm that exceeded 90% at 3%/3 mm for all gamma scores at the isocenter plane. The average gamma scores of the PCS were <90%. The dose differences in the homogeneous central region of the phantom were within ± 3% for all dose algorithms (Table 1). In contrast, AXPT showed dose errors exceeding 5% (up to 12.8%) within the lung rods, resulting in worse gamma scores than RayMC. Thus, RayMC achieved better dose calculation accuracy in heterogeneous regions than AXPT because RayMC could satisfy within ±5% for all densities.

Table 2 shows the optimization and final dose calculation times for the four dose calculation algorithms. With the use of GPUs, RayMC achieved the shortest dose calculation results for both the optimization and final calculations. Although the AXPT can be calculated using a GPU, this results in unacceptable dose calculation times for clinical use.

**TABLE 2:** Summary of optimization and final dose calculation time.

Optimization algorithms	Dose calculation algorithms	Optimization time (min)	Final dose calculation time (min)
NUPO	PBA	1.47	0.33 ± 0.10
NUPO	AXPT	1.47	160.77 ± 30.40
RayPBA	RayPBA	0.54	0.10 ± 0.01
RayMC	RayMC	0.37	0.09 ± 0.02

## 5 DISCUSSION

We performed preclinical commissioning of the most compact, newly scanned proton therapy system offered by Varian. In our hospital, the computational accuracy of four dose calculation algorithms for two different TPSs was performed using the same plan and compared with previous reports.^11^, ^12^, ^13^ This study differs from previous studies in that it used a proton‐beam device with a source‐to‐axis distance shorter than 200 cm, a GPU was used for MC, and the computational accuracy was evaluated using different RSs for the same phantom in patient validation.

Eclipse PCS Box plans, with and without RS, achieved >90% gamma scores at 3%/3 mm for all (Figure 3). However, with a gamma score of 2%/2 mm, numerous results below 90% were observed, particularly in areas with depths shallower than 100 mm. PCS cannot accurately calculate the halo component after RS passage.^27^ In addition, in‐air modeling parameters cannot fully capture scattering changes at high energies or during water penetration.^27^ No deviations were observed in the high‐energy region. Furthermore, the Eclipse PCS satisfied the acceptable limits for gamma scores and dose differences of at least a 3 × 3 cm^2^ irradiation field. This indicates that the field size factor correction was properly applied.^39^ The Eclipse registers a beam size that includes all RSs, resulting in a better gamma score and absolute dose agreement than RayPBA. RayPBA overestimated shallow/plateau doses, consistent with nuclear‐halo effects reported previously (Figure 2 and S3).^39^, ^40^ This effect was similar to the trend observed in the commissioning of RayStation with other proton beam systems.^12^, ^41^ Furthermore, Lin et al. demonstrated that for small fields and high‐energy (>200 MeV) proton beams, AXPT overestimates MCS and exaggerates the halo component, causing noticeable deviations in penumbra size and a decline in overall calculation accuracy.^13^ This results in known deterioration of computational accuracy under the aforementioned conditions. In this study, this tendency was observed in the high‐energy region using the Box plans. If an unacceptable gamma score is observed during dose verification, it is necessary to modify the algorithm or review beam parameters (such as gantry angle, range shifter, or air gap) to perform treatment using an acceptable beam.

For the mock patient plans, the PCS and RayPBA showed discrepancies between the calculations and measurements at several points compared to the RS. These measurements were improved using RayMC and AXPT, with RayMC being particularly accurate, with gamma scores exceeding 90% at 3%/3 mm for all, and the mean value achieving 95% of the guideline requirement of 3%/3 mm.^8^, ^29^ In a study by Langner et al., absolute dose differences within ± 3% were reported as acceptable values, which were best met by the RayMC.^11^ This is consistent with the trends observed in a previous study.^4^ As previously mentioned, Lin et al. also reported that the absolute dose agreements between the AXPT calculations and measurements tended to be overestimated in the deep (higher‐energy) region using the MCS and nuclear halo implementation, resulting in slightly larger dose differences.^13^ However, our results of the mock patient plans did not exhibit this trend because they did not use the high energies affected by AXPT. Instead, we confirmed the dose disagreements in beams using the RS, especially in mock patient beams (Figure S4). As RSs of three different thicknesses were available on this machine, we propose that treatment planning be performed with the thinnest RS thickness possible, depending on the target depth, in terms of dose agreements.

Although MC results can vary across runs at fixed statistical uncertainty, RayMC produced identical results across repeats in our tests (fixed seeds/parameters). The RayStation intentionally sets the initial random number seed to be identical, causing the MC method to produce identical results under the same conditions. This is a design choice by the vendor, addressing the user's expectation that identical MC calculations should yield identical results when implementing a treatment plan. Therefore, once the plan parameters (energies, spot positions, and spot MUs) and parameters such as the statistical error for MC calculations are determined, the calculation side will produce the specified calculation values. As such, the calculation and measurement results show the same trend each time. Regarding Eclipse's AXPT, its specification sets the initial random seed randomly, meaning this TPS shows different dose calculation results each time the calculation is performed. However, the reproducibility of the calculation results was extremely high, and it was considered unlikely that verification results would differ across different trials.

The heterogeneity results showed that the two MCs achieved better agreement with the calculations than the PCS and RayPBA calculations did. This trend is similar to that of previous studies on similar devices and confirms the importance of using MC in heterogeneity.^12^, ^42^ However, only RayMC showed absolute dose agreement within ± 5% in the lung region, suggesting that AXPT is limited in terms of its partial calculation accuracy. Therefore, RayMC is the most effective treatment option for patients with heterogeneous tumor regions. However, the quality of the final treatment plan deteriorates if the MC is not used for optimization in heterogeneous regions.^43^, ^44^ Based on these findings, RayMC should be used for both optimization and final calculations. Facilities that own both TPSs can use the PCS in homogeneous areas and RayMC in heterogeneous areas and use it as a calculation algorithm for secondary checks to improve safety in treatment planning. Our results indicate that heterogeneous areas can only be evaluated using RayMC, but the ability to perform secondary checks on relatively homogeneous areas and quality assurance applications, such as the abdomen and prostate, in all algorithms would be useful to users. This study could serve as a basis for examining these possibilities.

In addition to dose calculation accuracy, dose calculation time is a major factor in selecting a clinically usable dose calculation algorithm for TPS. Although RayMC is known to have excellent dose calculation accuracy, a significant increase in dose calculation time with the MC algorithm is an issue.^22^, ^23^ The current results indicate that RayMC is the most accurate and fastest method for calculating the dose for heterogeneous plans (Table 2). Therefore, we believe that RayMC is the most reasonable choice of calculation algorithm for clinical applications. Regarding GPUs, both RayStation 10A and Eclipse used the same NVIDIA GPU (Quadro P5000) specifications. The remaining factors affecting the computation time were mainly due to the MC algorithm implementation of each TPS. AXPT uses approximation methods (Section 2.2) in many areas compared to RayMC; however, the actual calculation time is significantly longer than that of RayMC. This is because of the algorithm, rather than the TPS hardware specifications. As mentioned earlier, RayMC produces the same calculation results regardless of the number of times the MC calculations are performed, provided that the parameters affecting the results are not altered. These timings suggest that RayMC transport is more streamlined than AXPT, yielding shorter computations. Users should be aware of the current status of these dose calculation times when using TPS systems in clinical practice. However, PBS planning is typically performed using robust optimization.^45^ Robust optimization is assumed to extend the optimization time.^46^ Furthermore, the final computation time is extended by increasing the target size. Although large targets were not considered in this study, both dose calculation accuracy and planning time are required to select appropriate algorithms for clinical applications. There may be situations where a less time‐consuming PBA or PCS is used that does not require such high precision.

This study has some limitations. First, the variety of targets in the box was extremely limited, and the plan using RS showed little variation in the air gaps. In addition, because the results were obtained from a study conducted before the start of treatment, we were unable to include the results of real patient validation in our study. However, there are no reports that comprehensively examine the four algorithms for the newest machines, and we believe that this study provides sufficient value. At our hospital, dose validation is conducted for each patient beam, and the results will be summarized and reported in a future study. We will be doing independent dose validations of this machine using our in‐house MC toolkit.^42^, ^47^, ^48^

## 6 CONCLUSIONS

We performed beam commissioning of four dose calculation algorithms from two commercial treatment planning systems on a proton therapy system newly released by Varian. The accuracy of the four calculations was generally acceptable compared with that of similar machines. However, the accuracy of the plans with RS deteriorated, especially for RayPBA. Both MC dose calculations in the TPSs showed higher accuracy than the analytical dose calculations in the heterogeneous region. Based on the dose validation results, Eclipse's PCS is a better choice than the PBA, whereas RayMC is more effective in situations where an MC is required. Given its accuracy and speed in this version, RayMC is the most appropriate engine across sites. These findings can support algorithm selection and commissioning strategies for newly installed compact PBS systems, as well as for the implementation of similar machines.

## AUTHOR CONTRIBUTIONS

Ryo Tanokura: Methodology, Data Curation, Formal analysis, Investigation, Writing—Review & Editing. Masato Horita: Methodology, Data Curation, Formal analysis, Investigation, Writing—Review & Editing. Makoto Matsumoto: Writing—Review & Editing. Akari Miyahara: Writing—Review & Editing. Shinji Furukawa: Writing—Review & Editing. Yasushi Ido: Writing—Review & Editing. Akira Matsumoto: Writing—Review & Editing. Shinichi Ogawa: Writing—Review & Editing. Wataru Makino: Writing—Review & Editing. Yoshito Matsui: Writing—Review & Editing. Yuki Tominaga: Conceptualization, Methodology, Software, Data Curation, Formal analysis, Investigation, Writing—Original Draft, Project administration. Takahiro Kato: Methodology, Data Curation, Formal analysis, Investigation, Writing—Review & Editing. Nobukazu Fuwa: Writing—Review & Editing.

## CONFLICT OF INTEREST STATEMENT

The authors declare that they have no competing financial or personal interests that may have influenced this study.

## Supporting information



Supporting Information
