# Supplementary material for: Beam commissioning of a new compact scanned proton therapy system with four different dose calculation algorithms
Source: J Appl Clin Med Phys. 2025 Dec 19;27(1):e70433. doi: 10.1002/acm2.70433 (PMC12715415; doi:10.1002/acm2.70433)
Supplement: Supplementary file 1 — Supporting Information [file ACM2-27-e70433-s001.docx]

**Supporting document**

​Figure S1. Screenshots of the v​alidation plans for (a) prostate SFO, (b) head and neck MFO, (c) C-shape MFO, and (d) heterogeneous plans. The cheese phantom of the heterogeneous plan had (e) four different rod-inserted areas present in front of the target.


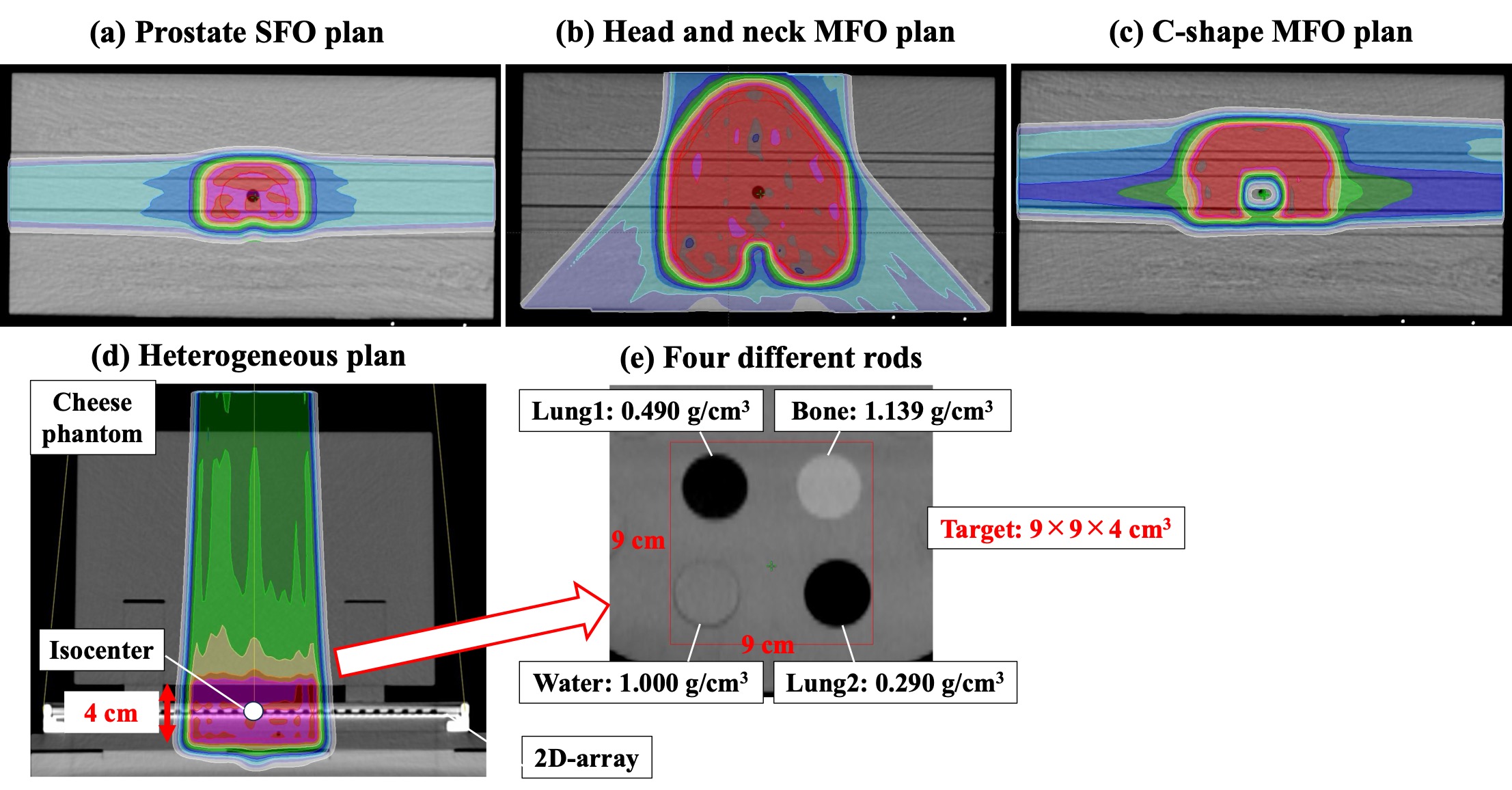


Abbreviations: MFO, multi-field optimization; SFO, single-field optimization

Figure S2. Computed Tomography table comparisons of (a) relative stopping power and (b) mass density using RayStation and Eclipse treatment planning systems (TPS).


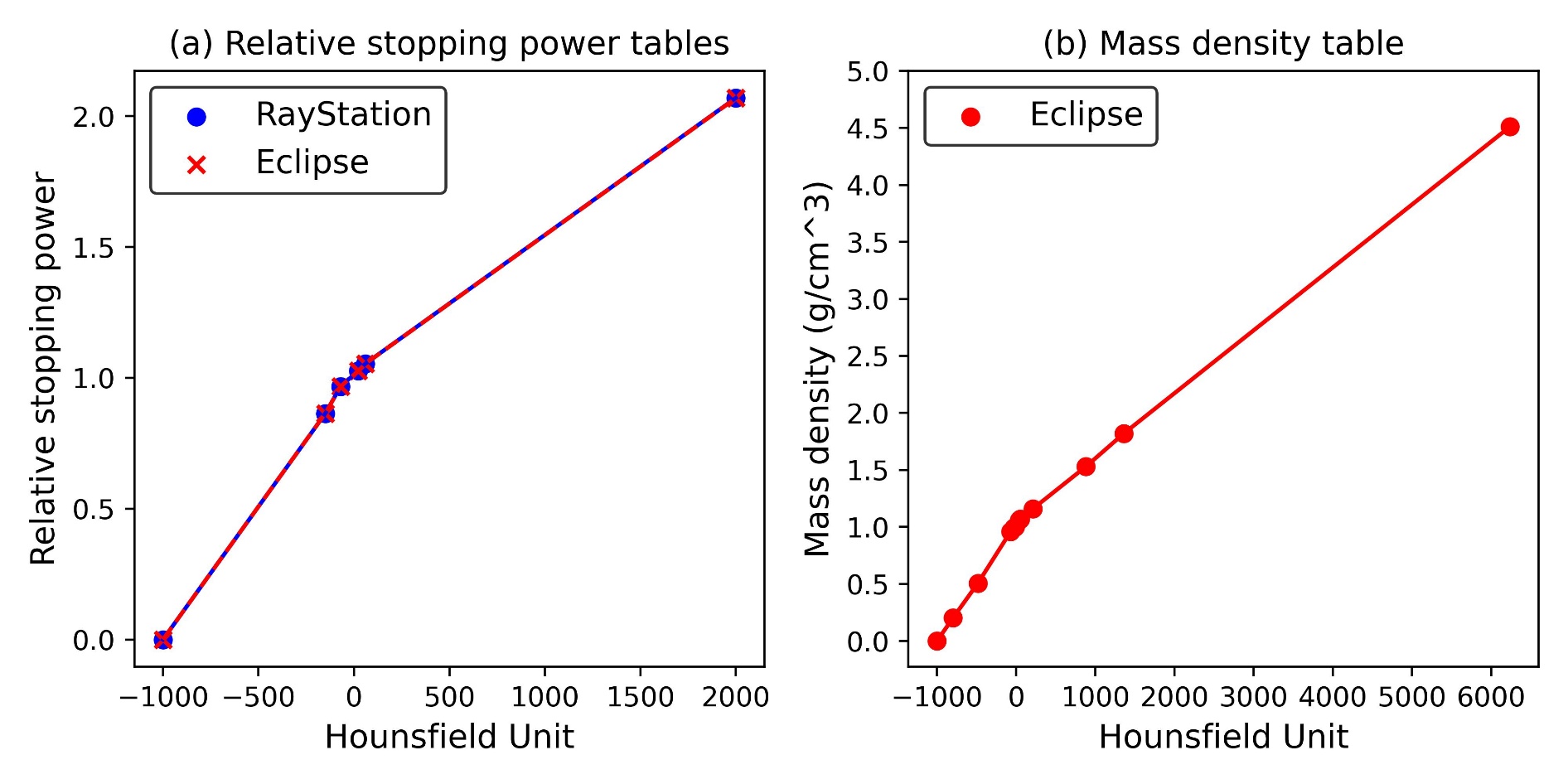


Figure S3. Three representative validation plan dose distributions (first column), depth doses of RayStation (second column) and Eclipse (third column), and lateral profiles of RayStation (fourth column) and Eclipse (fifth column) for (a) box with RS, (b) HN MFO, and (c) Prostate SFO plans, respectively. The black cross points indicate the isocenters. The white lines in the figure indicate the measured cross-sections for the depth doses and lateral profiles of the respective plans.


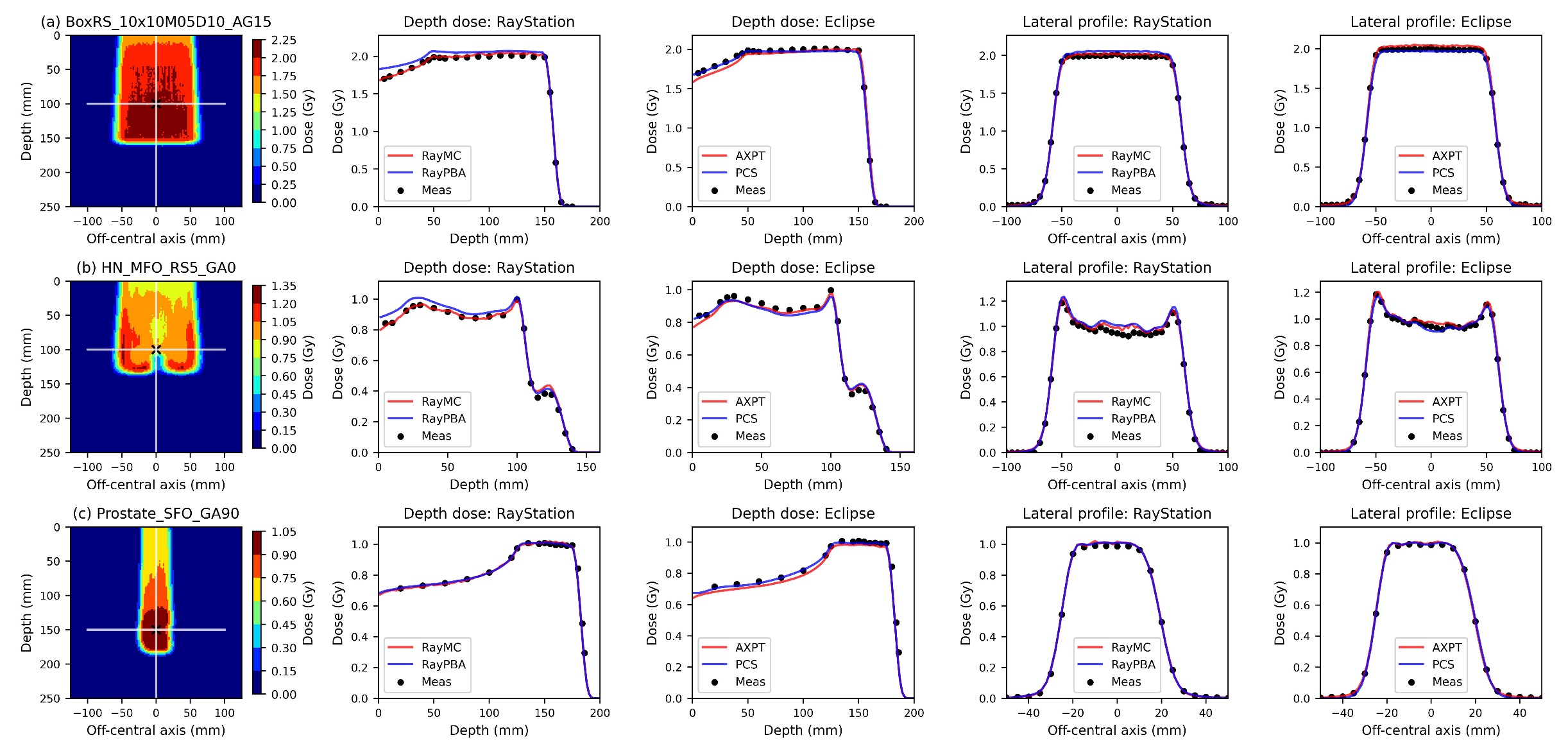


Figure S4. Scatter plots of dose differences in gamma scores of 2D dose measurements at 2%/2 mm (upper rows), 3%/3 mm (middle rows), and point doses at the isocenter planes (lower rows) for the four dose calculation algorithms at the measurement depth. The red lines in the figure indicate the 90% and ±5% tolerance values for the gamma scores and point doses, respectively.


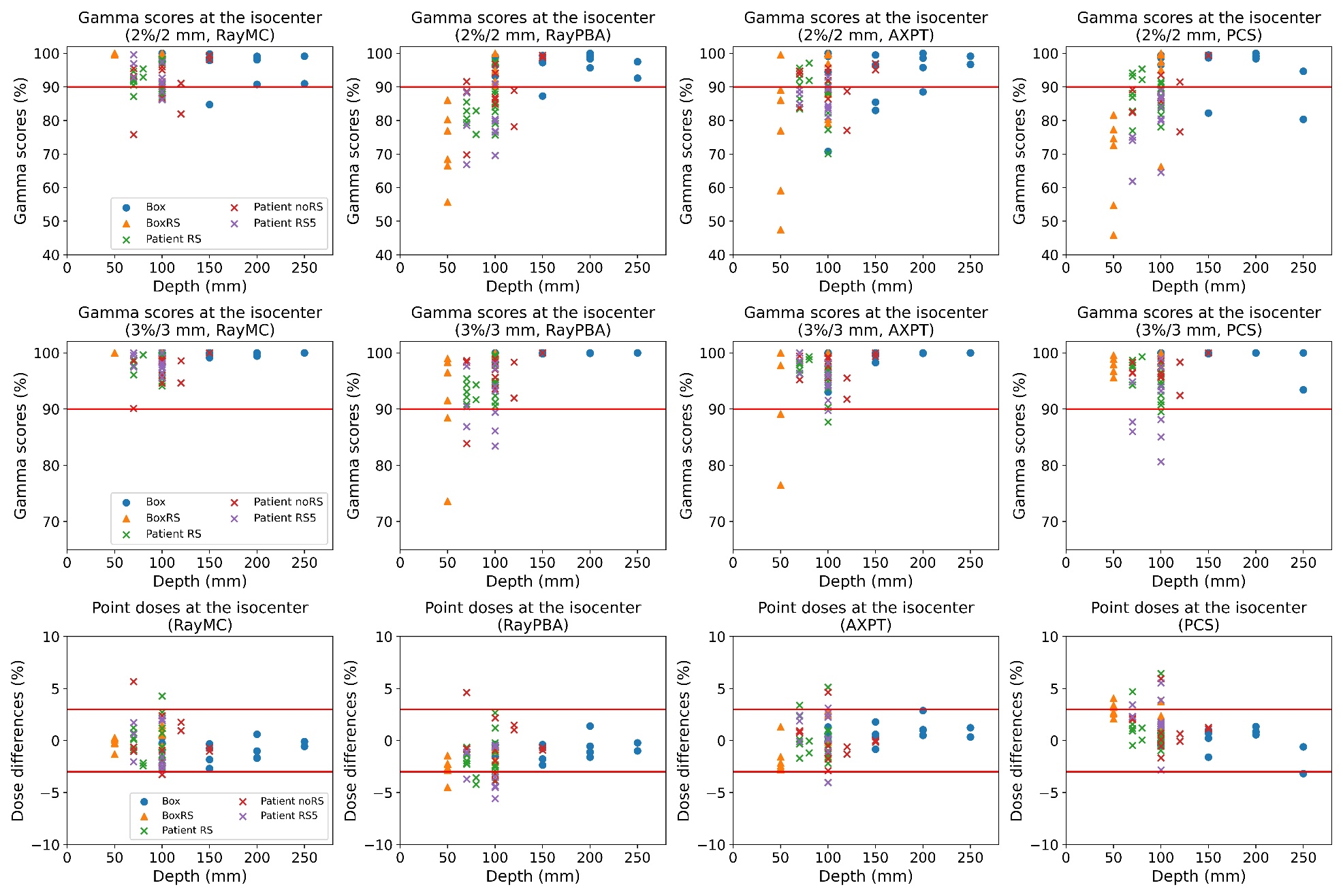


Figure S5. Scatter plots of dose differences in gamma scores of 2D dose measurements at 2%/2 mm (upper rows), 3%/3 mm (middle rows), and point doses at the isocenter planes (lower rows) for the four dose calculation algorithms in the irradiation area. The red lines in the figure indicate 90% and ±5% tolerance values for the gamma scores and point doses, respectively.


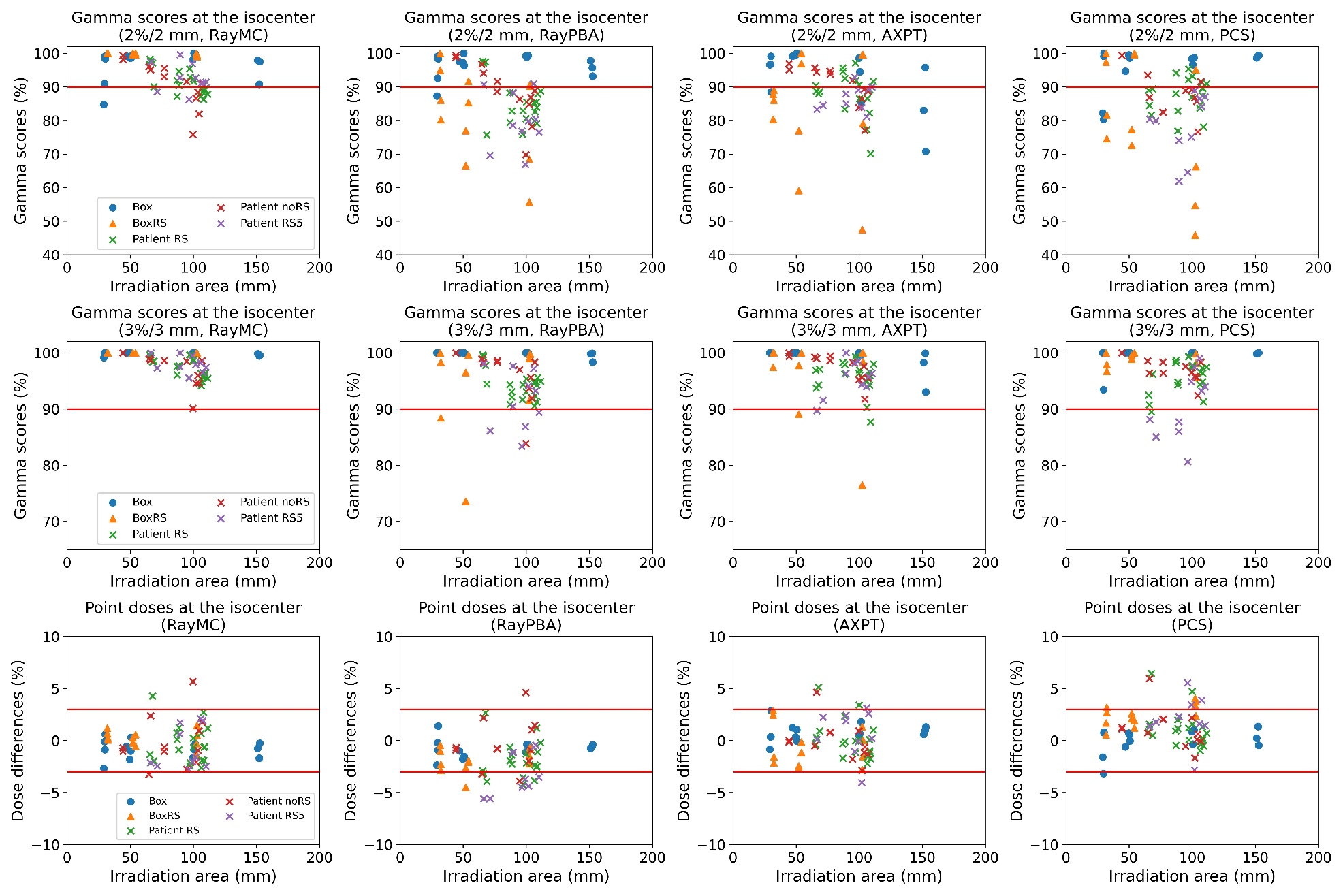


Figure S6. Validation results of heterogeneous plans with NUPO optimization for two different dose-calculation algorithms (RayPBA and PCS). The white lines in the dose and gamma-index distributions represent the cross-sections shown in the lateral profiles (x- and y-directions). PCS, proton convolution superposition


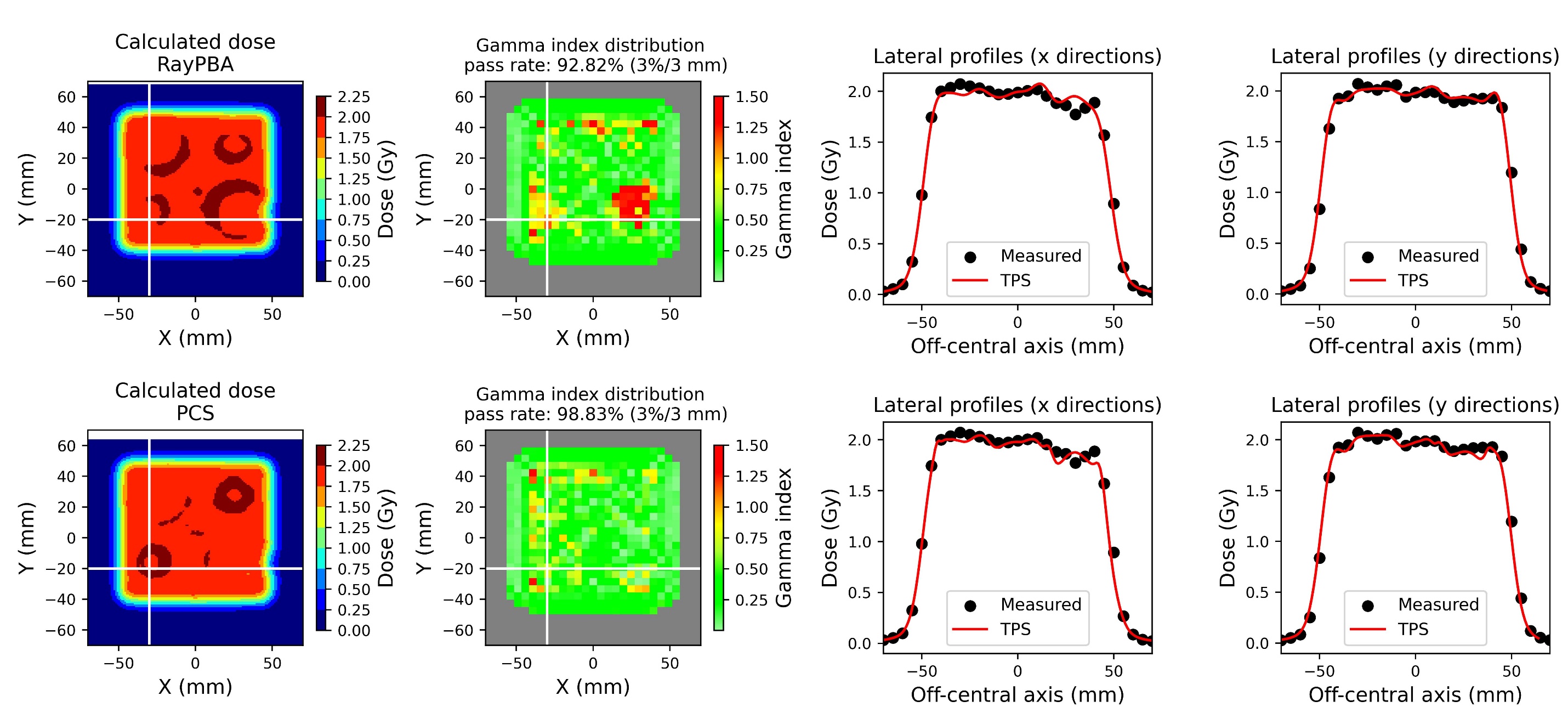


Table S1. TPS modeling data summary

| Energy (MeV) | Measured IDD R80 (Measured-NIST) [mm] | Eclipse IDD R80 (Eclipse-Measured) [mm] | RayPBA IDD R80 (RayPBA-Measured) [mm] | RayMC IDD R80 (RayMC-Measured) [mm] | Measured  spot size X (one sigma) [mm] | Eclipse spot size X (Eclipse-Measured) [mm] | RayPBA spot size X (RayPBA-Measured) [mm] | RayMC spot size X (RayMC-Measured) [mm] | Measured  spot size Y (one sigma) [mm] | Eclipse spot size Y (Eclipse-Measured) [mm] | RayPBA spot size Y (RayPBA-Measured) [mm] | RayMC spot size Y (RayMC-Measured) [mm] | Absolute dose calibration [GyRBE*mm^2^ /Spot MU] |
| --- | --- | --- | --- | --- | --- | --- | --- | --- | --- | --- | --- | --- | --- |
| 69 | 39.06 (-0.70) | 39.21 (0.14) | 39.14 (0.08) | 39.13 (0.07) | 5.67 | 5.65 (-0.02) | 5.70 (0.03) | 5.66 (-0.01) | 5.69 | 5.61 (-0.08) | 5.63 (-0.06) | 5.62 (-0.07) | 0.467 |
| 70 | 40.10 (-0.70) | 40.20 (0.10) | 40.16 (0.06) | 40.15 (0.06) | 5.62 | 5.59 (-0.03) | 5.67 (0.05) | 5.63 (0.01) | 5.65 | 5.58 (-0.07) | 5.62 (-0.03) | 5.59 (-0.06) | 0.462 |
| 80 | 51.19 (-0.65) | 51.36 (0.17) | 51.29 (0.10) | 51.25 (0.06) | 5.21 | 5.18 (-0.03) | 5.33 (0.12) | 5.28 (0.07) | 5.31 | 5.29 (-0.02) | 5.35 (0.04) | 5.25 (-0.06) | 0.427 |
| 90 | 63.54 (-0.44) | 63.72 (0.18) | 63.62 (0.08) | 63.63 (0.09) | 4.95 | 4.91 (-0.04) | 5.08 (0.13) | 5.04 (0.09) | 5.06 | 5.04 (-0.02) | 5.11 (0.05) | 5.01 (-0.05) | 0.407 |
| 100 | 76.76 (-0.42) | 76.96 (0.21) | 76.85 (0.09) | 76.80 (0.05) | 4.73 | 4.68 (-0.05) | 4.87 (0.14) | 4.83 (0.10) | 4.85 | 4.83 (-0.02) | 4.93 (0.08) | 4.81 (-0.04) | 0.395 |
| 110 | 91.07 (-0.33) | 91.24 (0.17) | 91.16 (0.09) | 91.10 (0.03) | 4.57 | 4.51 (-0.06) | 4.72 (0.15) | 4.68 (0.11) | 4.71 | 4.7 (-0.01) | 4.79 (0.08) | 4.66 (-0.05) | 0.387 |
| 120 | 106.26 (-0.34) | 106.45 (0.19) | 106.23 (-0.03) | 106.34 (0.08) | 4.42 | 4.35 (-0.07) | 4.59 (0.17) | 4.55 (0.13) | 4.57 | 4.55 (-0.02) | 4.69 (0.12) | 4.53 (-0.04) | 0.382 |
| 130 | 122.43 (-0.37) | 122.58 (0.15) | 122.53 (0.11) | 122.46 (0.03) | 4.28 | 4.21 (-0.07) | 4.47 (0.19) | 4.43 (0.15) | 4.45 | 4.44 (-0.01) | 4.6 (0.15) | 4.41 (-0.04) | 0.378 |
| 140 | 139.59 (-0.21) | 139.69 (0.10) | 139.57 (-0.02) | 139.57 (-0.02) | 4.18 | 4.10 (-0.08) | 4.37 (0.19) | 4.34 (0.16) | 4.35 | 4.33 (-0.02) | 4.51 (0.16) | 4.31 (-0.04) | 0.375 |
| 150 | 157.51 (-0.19) | 157.56 (0.05) | 157.51 (0.00) | 157.51 (0.00) | 4.09 | 4.00 (-0.09) | 4.27 (0.18) | 4.24 (0.15) | 4.25 | 4.23 (-0.02) | 4.42 (0.17) | 4.21 (-0.04) | 0.374 |
| 160 | 176.37 (-0.13) | 176.37 (0.00) | 176.41 (0.05) | 176.35 (-0.01) | 4.01 | 3.90 (-0.11) | 4.18 (0.17) | 4.15 (0.14) | 4.16 | 4.12 (-0.04) | 4.33 (0.17) | 4.12 (-0.04) | 0.374 |
| 170 | 196.10 (0.00) | 196.11 (0.01) | 196.08 (-0.02) | 196.09 (-0.01) | 3.94 | 3.82 (-0.12) | 4.08 (0.14) | 4.05 (0.11) | 4.05 | 3.99 (-0.06) | 4.18 (0.13) | 4.03 (-0.02) | 0.374 |
| 180 | 216.44 (-0.06) | 216.40 (-0.04) | 216.39 (-0.05) | 216.44 (0.00) | 3.89 | 3.76 (-0.13) | 4.04 (0.15) | 4.02 (0.13) | 4.01 | 3.93 (-0.08) | 4.18 (0.17) | 3.99 (-0.02) | 0.374 |
| 190 | 237.62 (-0.08) | 237.53 (-0.09) | 237.63 (0.01) | 237.61 (-0.01) | 3.88 | 3.72 (-0.16) | 4.04 (0.16) | 4.01 (0.13) | 4.01 | 3.93 (-0.08) | 4.13 (0.12) | 3.98 (-0.03) | 0.376 |
| 200 | 259.45 (-0.15) | 259.33 (-0.12) | 259.4 (-0.05) | 259.43 (-0.02) | 3.88 | 3.69 (-0.19) | 4.02 (0.14) | 4.00 (0.12) | 3.99 | 3.91 (-0.08) | 4.11 (0.12) | 3.97 (-0.02) | 0.377 |
| 210 | 282.05 (-0.15) | 281.96 (-0.09) | 282.08 (0.03) | 282.02 (-0.04) | 3.89 | 3.68 (-0.21) | 4.02 (0.13) | 4.00 (0.11) | 3.99 | 3.9 (-0.09) | 4.06 (0.07) | 3.97 (-0.02) | 0.378 |
| 218 | 300.42 (-0.38) | 300.34 (-0.08) | 300.46 (0.04) | 300.43 (0.01) | 3.95 | 3.71 (-0.24) | 4.04 (0.09) | 4.02 (0.07) | 4.01 | 3.86 (-0.15) | 4.1 (0.09) | 3.99 (-0.02) | 0.379 |

Abbreviations: NIST, National Institute of Standards and Technology Proton Stopping Power and Continuous Slowing Down Approximation (CSDA) Range; IDD, integrated depth dose; R80, the range defined by 80% of the maximum value of a Bragg peak; RayMC, RayStation Monte Carlo algorithm; TPS, treatment planning systems

Table S2. Validation plan summary. Depth doses were measured only for the plans with bold numbers in table.
The X and Y in the plan names “X”×”X”_M”Y” indicate the target irradiation field (cm) and modulation width (cm), respectively.

| Plan type | Plan name | Depth (cm) | Air gap (cm) | Range shifter (mm) | Dose (GyRBE) | Gantry angles (degree) | Number of fields | Number of  depth dose measurements | Number of  2D dose measurements |
| --- | --- | --- | --- | --- | --- | --- | --- | --- | --- |
| Cubic targets (Box) | 03×03_M04 | **10, 15, 20, 25** | - | None | 2.0 | 0 | 4 | 4 | 4 |
|  | 05×05_M06 | **10, 15, 20, 25** | - | None | 2.0 | 0 | 4 | 4 | 4 |
|  | 10×10_M10 | 10, **15,** 20 | - | None | 2.0 | 0 | 3 | 1 | 3 |
|  | 15×15_M10 | 10, **15**, 20 | - | None | 2.0 | 0 | 3 | 1 | 3 |
| Cubic targets with range shifter (BoxRS) | 03×03_M05 | 5 | **5.0, 15.0** | 50 | 2.0 | 0 | 2 | 2 | 2 |
|  | 03×03_M10 | 10 | **5.0, 15.0** | 50 | 2.0 | 0 | 2 | 2 | 2 |
|  | 03×03_M05 | 5 | **5.0, 15.0** | 50 | 2.0 | 0 | 2 | 2 | 2 |
|  | 03×03_M10 | 10 | **5.0, 15.0** | 50 | 2.0 | 0 | 2 | 2 | 2 |
|  | 10×10_M05 | 5 | **5.0, 15.0** | 50 | 2.0 | 0 | 2 | 2 | 2 |
|  | 10×10_M10 | 10 | **5.0, 15.0** | 50 | 2.0 | 0 | 2 | 2 | 2 |
| Mock patients  (AAPM TG-119) | Prostate | 15 | - | **None** | 2.0 (plan dose) | 90, 270 | 2 (2 fields/plan) | 2 | 2 |
|  | HN MFO | 7-12 | 15.0 | **None**, 20, 30, **50** | 2.0 (plan dose) | 0, 135, 225 | 12 (3 fields/plan) | 6 | 12 |
|  | HN SFO | 7-10 | 15.0 | None, 20, 30, 50 | 2.0 (plan dose) | 0, 135, 225 | 12 (3 fields/plan) | - | 12 |
|  | Cshape MFO | 10 | 15.0 | **None**, 20, 30, **50** | 2.0 (plan dose) | 90, 270 | 8 (2 fields/plan) | 4 | 8 |
|  | Cshape SFO | 7 | 15.0 | None, 20, 30, 50 | 2.0 (plan dose) | 45, 315 | 8 (2 fields/plan) | - | 8 |
| Heterogeneous | Cheese RayMC | Phantom isocenter | - | None | 2.0 | 0 | 3 | - | 1 |
|  | Cheese RayPBA | Phantom isocenter | - | None | 2.0 | 0 | 3 | - | 1 |
|  | Cheese AXPT | Phantom isocenter | - | None | 2.0 | 0 | 3 | - | 1 |
|  | Cheese PCS | Phantom isocenter | - | None | 2.0 | 0 | 3 | - | 1 |

Abbreviations: HN, head and neck; MFO, multi-field optimization; SFO, single-field optimization; Cheese, tomotherapy cheese phantom, RayMC, RayStation Monte Carlo algorithm; RayPBA, RayStation pencil beam algorithm; AXPT, Eclipse AcurosPT algorithm; PCS, Eclipse proton convolution superposition algorithm
